# Prolonged inhibition of class I PI3K promotes liver cancer stem cell expansion by augmenting SGK3/GSK-3β/β-catenin signalling

**DOI:** 10.1186/s13046-018-0801-8

**Published:** 2018-06-25

**Authors:** Fengchao Liu, Xiaoling Wu, Xin Jiang, Yanzhi Qian, Jian Gao

**Affiliations:** Department of Gastroenterology, Second Affiliated Hospital, Chongqing Medical University, Chongqing, China

**Keywords:** Cancer stem cells, HCC, SGK3, PI3K, GSK-3β/β-catenin signalling pathway

## Abstract

**Background:**

Serum and glucocorticoid-regulated kinase 3 (SGK3) has been reported to play an important role in tumour progression, but its role in cancer stem cells (CSCs) remains obscure. The phosphoinositide 3-kinase (PI3K) pathway is considered a hallmark of cancer. Although many PI3K pathway-targeted therapies have been tested in oncology trials, the results are not satisfactory.

**Methods:**

We used spheroids cultured in serum-free culture medium and MicroBead isolation to obtain liver CSCs. Spheroid formation assay and flow cytometric analysis were performed to investigate liver CSC expansion. Real-time polymerase chain reaction (PCR), western blot and immunofluorescence were used to assess gene expression in cell lines.

**Results:**

We found that SGK3 is preferentially activated in liver CSCs. Upregulated SGK3 significantly increases the expansion of liver CSCs. Conversely, suppression of SGK3 in human hepatocarcinoma (HCC) cells had an opposite effect. Mechanistically, SGK3 promoted β-catenin accumulation by suppressing GSK-3β-mediated β-catenin degradation in liver CSCs, and then promoting the expansion of liver CSCs. Prolonged treatment of HCC cells with class I PI3K inhibitors leads to activation of SGK3 and expansion of liver CSCs. Inhibition of hVps34 can block SGK3 activity and suppress liver CSC expansion induced by PI3K inhibitors. More importantly, we also found that prolonged treatment of HCC cells with PI3K inhibitors stimulates the β-catenin signalling pathway via activation of SGK3.

**Conclusions:**

Prolonged inhibition of class I PI3K promotes liver CSC expansion by augmenting SGK3-dependent β-catenin stabilisation, and effective inhibition of SGK3 signalling may be useful in eliminating liver CSCs and in PI3K pathway-targeted cancer therapies.

**Electronic supplementary material:**

The online version of this article (10.1186/s13046-018-0801-8) contains supplementary material, which is available to authorized users.

## Background

Hepatocellular carcinoma (HCC) is one of the leading causes of cancer-related death and is the main severe consequence leading to death in patients with cirrhosis and many other chronic liver diseases [1, 2]. Despite recent progress in HCC treatment, prognosis for this refractory disease remains unsatisfactory [3] because both solid tumours show considerable histological and functional heterogeneity [4]. Such cellular heterogeneity is very important due to its important role in treatment resistance. Recent studies have suggested that subpopulations of cells with increased tumorigenesis capacities and self-renewal potential, termed as cancer stem cells (CSCs) [5], exist within tumours. Persistence of CSCs is a primary cause of relapse and metastasis, which are highly resistant to chemotherapy [6]. Therefore, more effective therapeutic strategies may be developed if the molecular mechanism underlying CSC regulation is illuminated.

The existence of CSCs has been demonstrated in a variety of solid tumours, including liver cancer [7]. Liver CSCs can be enriched with several defined surface markers, including CD133, CD90, CD44, OV6, EpCAM, CD13, CD24, ICAM-1, CD47, Lgr5, and keratin19 [8]. Although CSCs can be identified within the liver cancer cells, they cannot be effectively eradicated because the detailed regulatory mechanism of CSC generation and expansion remains largely unknown. Signalling pathways such as the Wnt/β-catenin, TGFβ, IL-6/STAT3, Notch and ANXA3/JNK pathways have been reported to be involved in the regulation of liver CSCs [9–12]. Among these pathways, Wnt/β-catenin signalling has received increasing attention because of its important role in both normal stem cells and CSCs. Inhibition of the Wnt/β-catenin pathway has also been shown to be effective in eliminating CSCs [13]. However, the deregulation of Wnt/β-catenin pathway in liver CSCs is not fully understood.

The phosphoinositide 3-kinase (PI3K) pathway is a very important intracellular signalling pathway, which plays crucial roles in normal cell processes and a critical role in cancers. Several studies have explored the therapeutic targeting of the PI3K pathway in cancers, and various inhibitors targeting PI3K and its isoforms have been developed [14]; however, the clinical effect was not satisfactory. The role of the PI3K signalling pathway in CSCs has been reported, but some controversy remains [15].

Serum and glucocorticoid-regulated kinase 3 (SGK3), an AGC protein kinase family member, has been found to play a critical role in a variety of cancers [16]. A previous study showed that PIK3CA-mediated breast cancer cell growth and survival are dependent on the SGK3, and Akt is dispensable [17]. SGK3 is a unique member of the SGK family because it contains an N-terminal PX domain. SGK3 binds selectively to PtdIns(3)P through its PX domain, which is required for targeting SGK3 to the endosome, where the Class III PI3K (also termed hVps34) phosphorylates PtdIns to generate a pool of PtdIns(3)P [18, 19]. VPS34-IN1, an hVps34 inhibitor can suppress SGK3 activation by reducing PtdIns(3)P levels via lowering phosphorylation of T-loop and hydrophobic motifs [20, 21]. Amplification and overexpression of SGK3 have been reported more frequently than those for AKT in HCC, suggesting it may have a greater functional significance in HCC [22]. Our previous study found that SGK3 plays an important role in the invasive potential of HCC cells and epithelial-mesenchymal transition (EMT) [23]. However, the role and mechanism of SGK3 in CSCs has not been reported.

Here, we show that SGK3 is preferentially activated in liver CSCs, and upregulated or downregulated SGK3 in HCC cells enhances or suppresses liver CSC-associated gene expression and spheroid formation via the GSK-3β/β-catenin signalling pathway. We also found that prolonged treatment of HCC cells with PI3K inhibitors leads to activation of SGK3 and expansion of liver CSCs. Additionally, our results demonstrate that inhibition of hVps34 can block SGK3 activity and suppress liver CSC expansion. Effective inhibition of SGK3 signalling may be useful in eliminating liver CSCs.

## Methods

### Cell lines and culture

The Huh7 cells were obtained from the Chinese Academy of Sciences Cell Bank. The MHCC-97H cells were obtained from Zhongshan Hospital of Fudan University in Shanghai, China. All cells were maintained in Dulbecco’s modified Eagle medium (DMEM; Heclone) supplemented with 10% foetal bovine serum (FBS; Capricorn). Liver CSCs were cultured in serum-free culture medium. Serum-free culture medium was DMEM/F12 (Hyclone) consisting of 20 ng/ml basic fibroblast growth factor (bFGF; PeproTech), 20 ng/ml epidermal growth factor (EGF; PeproTech) and 20 μl/ml B27 supplement (Life Technologies).

### Isolation of CD133+ cells

CD133+ cells were obtained using the CD133 MicroBead Kit (Miltenyi Biotec) according to the manufacturer’s instructions. HCC cells were enzymatically dissociated, the cell suspension was centrifuged at 300×g for 10 min, and the supernatant was aspirated completely. The cells were resuspended in 300 μl of buffer per 10^8^ cells, and 100 μl of FcR Blocking Reagent and 100 μl of CD133 MicroBeads were added, mixed well and incubated for 30 min at 4 °C. Then, the cells were washed by adding 1 ml of buffer, centrifuged at 300×g for 10 min, and sorted with the Mini MACS® Separator (Miltenyi Biotec). Phycoerythrin (PE)-conjugated CD133/2 antibodies (Miltenyi Biotec) were used to evaluate the efficiency of magnetic separation by flow cytometry.

### RNA extraction and quantitative reverse transcription polymerase chain reaction (RT-PCR)

Total RNA was extracted from the cells by using Trizol (Invitrogen). Complementary DNA (cDNA) synthesis was performed using the PrimeScript™ RT Reagent Kit with gDNA Eraser (Takara). Quantitative RT-PCR was performed using the SYBR Premix ExTaq (Takara) under standard conditions according to the manufacturer’s instructions. Quantitative RT-PCR was conducted with the CFX96 Real-Time PCR Detection System (Bio-Rad). The data were analysed using the 2^−△△Ct^ method. The primers are listed in Additional file 1: Table S1.

### Cell siRNA transfection and inhibitors

Small interfering RNAs (siRNA) of SGK3 and negative control (NC) were designed and synthesised by RiboBio (Guangzhou, China). The cells were transfected using a ribo FECT™ CP Transfection Kit (RiboBio) according to the manufacturer’s protocol. A total of 2 × 10^5^ cells were seeded per well and grown to 50–70% confluence. Transfection complexes were prepared according to the instructions and were added directly to the cells. The siRNA and NC were used at a final concentration of 100 nM. The mRNA level was detected by RT-PCR after incubation for 24 h, and the level of protein was determined by western blot after incubation for 48 h. Class I PI3K inhibitors ZSTK474 and LY294002, hVps34 inhibitor VPS34-IN1 and GSK 3β inhibitor AR014418 were purchased from MedChem Express (MCE).

### Establishment of the SGK3 stable overexpression and knockdown cell lines

To establish stable transduction, lentiviral vectors expressing SGK3 sequence, shRNA and the control vectors were obtained from Hanbio (Shanghai, China). shRNA-mediated silencing of SGK3 required the synthesis of a set of oligonucleotides composed of a target shRNA sequence and its complement against SGK3, as previously described [24]. Polybrene (Hanbio) was used to promote the transfection according to the manufacturer’s instruction. On the previous day, 1 × 10^5^ cells were seeded per well and grown to 20–40% confluence. Then, the cells were transfected at a multiplicity of infection (MOI) of 20. After 72 h, the transfection efficiency was verified by fluorescence microscopy and RT-PCR.

### Spheroid formation assay

The cell spheroid formation assay was performed as described previously [10]. Briefly, single cells (1 × 10^3^) were plated in a 6-well ultra-low attachment plate (Corning) or (1 × 10^2^) in a 24-well ultra-low attachment plate in the serum-free culture medium. After 1–2 weeks, the number of tumour spheroids (diameter > 50 μm) was counted under an inverted microscope.

### Protein extraction and western blotting

Western blot analysis to determine protein level was performed as described previously [10]. The following antibodies were used: anti-SGK3 (sc-166,847; Santa Cruz), anti-β-actin (YT0099; Immunoway), anti-Akt1 (ab32505; Abcam), anti-Akt1 (phosphoS473; ab81283; Abcam), anti-SGK3 (phosphoThr320; #5642; CST), anti-GSK3β (ab32391, Abcam), anti-GSK3β (phosphoS9; ab75814; Abcam), anti-β-catenin (#8480, CST), anti-CD133 (YT5192; Immunoway) and anti-Nanog (YM0464; Immunoway).

### Immunofluorescence

Cell slides were fixed with 4% paraformaldehyde for 20 min, and permeabilised with 0.3% Triton X-100 (Sigma-Aldrich) for 15 min. Then, the cells were blocked with normal goat serum and incubated with anti-Nanog (1:100; Immunoway) at 4 °C overnight, followed by incubated with Alex 555-conjugated goat anti-mouse antibody for 1 h. The cells were counterstained with 4′, 6-diamidino-2-phenylindole (DAPI) for 5 min and visualised by fluorescent microscope (Nikon).

### Immunohistochemical assay

Tumour tissues from the nude mice were fixed in 4% formaldehyde for 24 h, embedded in paraffin, and serially sectioned at a thickness of 6 μm. Sections were deparaffinized and stained with anti-CD133 (1:500; ab222782; Abcam) at 4 °C overnight, and incubated with the secondary antibody for 1 h at 37 °C. Reaction results were shown by incubation with 3, 3′-Diaminobenzidine (DAB; Boster). After washing with tap water to stop the chromogenic reaction, the sections were dehydrated in an ascending alcohol gradient, cleared twice with xylene and mounted in neutral balsam. Then, the sections were examined and imaged by microscope (Nikon).

### Flow cytometric analysis

Cells were collected and resuspended in 100 μl of phosphate-buffered saline (PBS) containing 20 μl FcR Blocking Reagent (Miltenyi Biotec) and anti-human antibodies, PE-CD133 (Miltenyi Biotec), and then incubated for 15 min on ice in the dark. After incubating, the cells were washed twice with 1 ml of PBS. The collected cells were resuspended in 300 μl of PBS and detected using a FACSCanto II flow cytometer (BD Biosciences). Isotype-matched mouse antibodies served as controls.

### In vivo xenograft experiments

All animal experiments were performed in compliance with the strict rules of the Animal Ethics Committee of Chongqing Medical University. For tumour formation assay, 1 × 10^4^ CD133+/− cells were subcutaneously injected into 6-week-old female athymic nude mice. Tumour formation was observed every week and analysed at the sixth week. The effect of ZSTK474 was tested in vivo. MHCC97H cells were subcutaneously injected into 6-week-old female athymic nude mice (5 × 10^6^ cells per mouse) and allowed to form tumours. Once the tumours reached 300 mm^3^, 6 animals were randomly divided into control and ZSTK474 groups. The ZSTK474 group was orally administered a dose of ZSTK474 (suspended in 5% hydroxypropyl cellulose) at 400 mg/kg daily for 10 days. The control group of mice was orally administered with 5% hydroxypropyl cellulose instead of ZSTK474. Tumours were measured throughout the treatment period.

### Statistical analysis

Statistical analyses were performed using SPSS 21 software. All data were acquired from at least 3 independent experiments and are reported as the mean ± SD. Two independent group comparisons were analysed using Student’s t-test. *P* < 0.05 was considered statistically significant.

## Results

### SGK3 is preferentially activated in liver CSCs

**Fig. 1:**
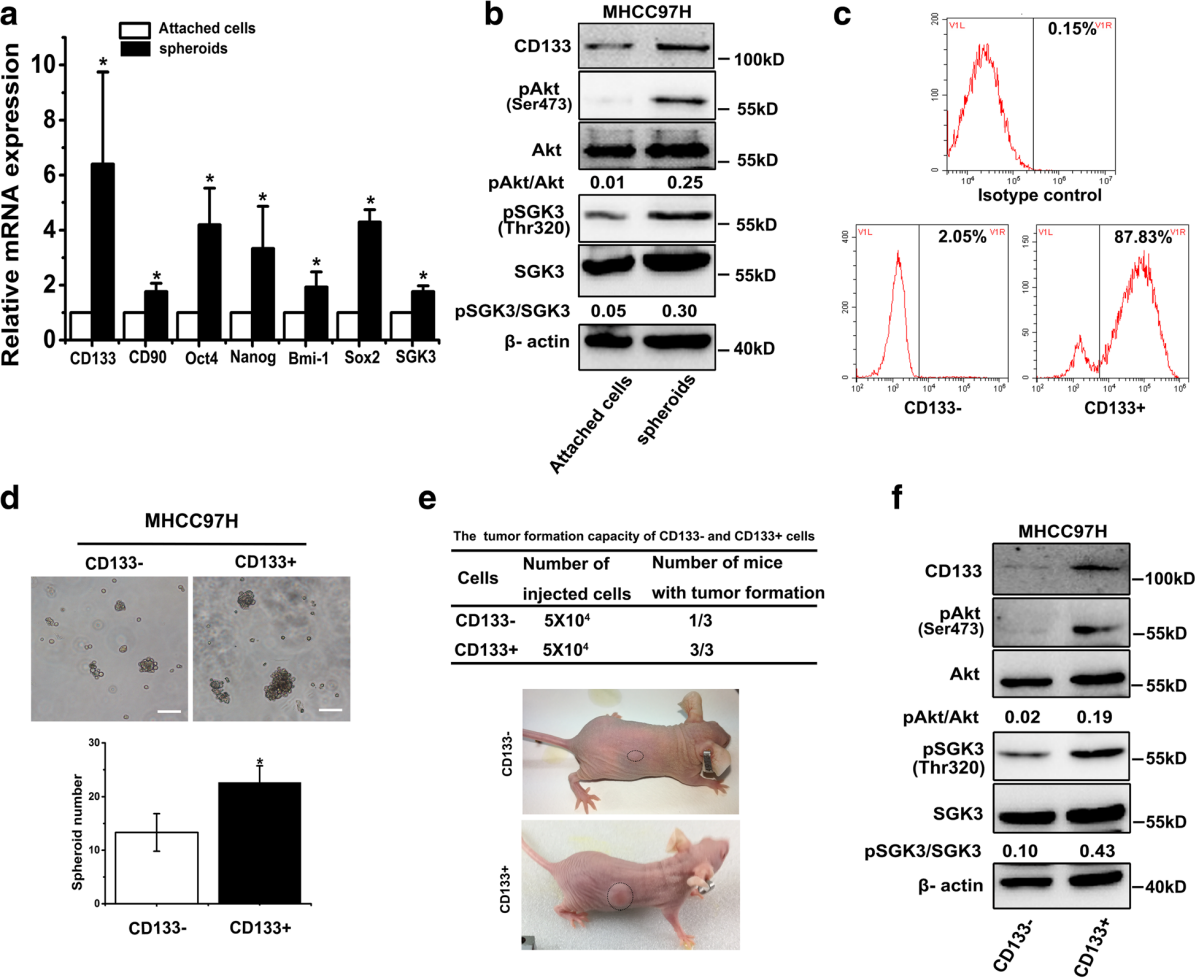
SGK3 is preferentially activated in liver CSCs. **a** MHCC-97H cells were cultured in monolayer or ultra-low attachment conditions. The mRNA expression of liver CSC-related genes and SGK3 in spheroids and attached cells was compared by RT-PCR. **b** Western blot analysis for levels of CD133, active Akt (phosphorylated at Ser473)/total Akt and active SGK3 (phosphorylated at Thr320)/total SGK3 between spheroids and attached cells. **c** Flow cytometry analysis of CD133+ cell distribution in CD133- and CD133+ cells isolated using CD133 MicroBead Kit. **d** Representative images of CD133+ and CD133- cells sorted from MHCC97H HCC cells cultured in serum-free culture medium after 7 days. Scale bars, 100 μm. **e** CD133+/CD133- cells were subcutaneously injected into 6-week-old female athymic nude mice, and tumourigenicity was evaluated 5 weeks post-inoculation. **f** Levels of CD133, active Akt (phosphorylated at Ser473)/total Akt and active SGK3 (phosphorylated at Thr320)/total SGK3 were compared by western blot analysis between CD133+ and CD133- cells. β-actin was used as a loading control. All experiments were performed in triplicate. *P < 0.05

It has been confirmed that the liver cancer cell spheroids cultured in serum-free culture medium are highly enriched in liver CSCs. To explore the relevance between SGK3 and liver CSCs, we detected expression levels of SGK3 mRNA in CSC-enriched hepatoma spheroids compared with monolayer-attached cells. The results showed that the mRNAs of several stem cell markers, including CD90, CD133, Oct4, Nanog, Bmi-1, and SOX2 were all upregulated in the spheroids compared to the monolayer-attached cells, and moderately elevated expression of SGK3 mRNA was observed in the spheroids (Fig. 1a). We next analysed SGK3 protein levels and phosphorylated levels in spheroids, and we found elevated SGK3 phosphorylation in spheroids, while no difference was observed between the expression of the total SGK3 protein in spheroids and attached cells. Consistently, the phosphorylation of Akt rather than total Akt proteins was elevated in spheroids (Fig. 1b).

CD133 is widely used as liver CSCs markers [25]. We then sorted CD133+ cells from MHCC97H HCC cells. After sorting, the purity of the selected CD133+ population was monitored by flow-cytometric analysis to ensure the positivity of CD133 was over 85% (Fig. 1c). After cultured in serum-free culture medium for 7 days, the number of spheroids (diameter > 50 μm) in the CD133+ cells was significantly higher than that in the CD133- cells (Fig. 1d). Furthermore, CD133+ cells displayed much higher tumour-initiating capacity in in vivo experiments (Fig. 1e). To further investigate the role of SGK3 in liver CSCs, we determined the phosphorylation of SGK3 in CD133+ and CD133- cells. As expected, phosphorylation of SGK3 in CD133+ HCC cells was significantly higher than that in CD133- cells (Fig. 1f). In addition, the phosphorylation of Akt rather than total Akt proteins was elevated in CD133+ HCC cells (Fig. 1f), which is consistent with findings reported by Ma et al. [26]. Taken together, these results indicate that SGK3 may play a crucial role in the expansion of liver CSCs.

### SGK3 enhances the expansion of liver CSCs

**Fig. 2:**
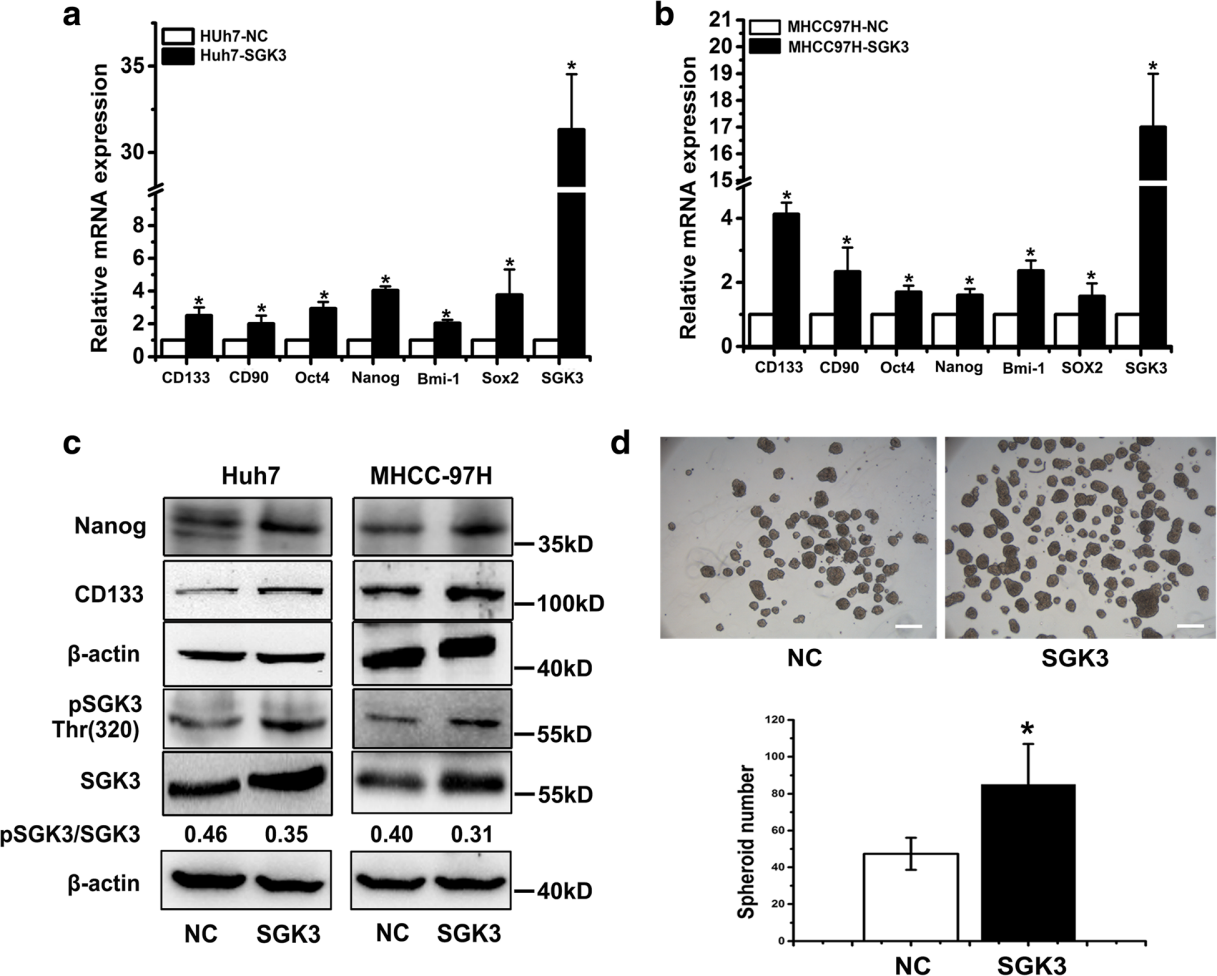
SGK3 enhances the expansion of liver CSCs. **a, b** Relative mRNA expression of liver CSC-related markers CD133, CD90, Nanog, Oct4, Bmi-1 and Sox2 in Huh7 and MHCC-97H cells stably overexpressing SGK3 or NC. β-actin was used as an loading control. **c** Expression of CD133, Nanog and SGK3 in Huh7 and MHCC-97H cells stably overexpressing SGK3 or NC detected by western blot. β-actin was used as a loading control. **d** Spheroid formation assay of MHCC97H-SGK3 or MHCC97H-NC cells (top). Scale bars, 200 μm. The statistical results of the tumour spherosis (> 50 μm) were calculated based on 3 independent experiments (bottom). All experiments were performed in triplicate, and the results are shown as mean ± standard deviation. *P < 0.05

To assess the role of SGK3 in liver CSC regulation, we established a lentivirus-mediated stable SGK3 or negative control (NC) expression cell lines using MHCC-97H and Huh7. Notably, SGK3 overexpression significantly enhanced the mRNA expression of CSC-related genes CD133, CD90, Oct4, Nanog, Bmi-1 and Sox2 compared with NC cells (Fig. 2a and b). Consistently, the expression of CD133 and Nanog proteins increased with the increase of SGK3 (Fig. 2c). To further elucidate the role of SGK3 in CSCs, we examined spheroid-forming ability (a surrogate marker of CSCs self-renewal). We found that SGK3 overexpression increased spheroid formation ability compared with NC cells (Fig. 2d). These results suggest that SGK3 enhances the expansion of liver CSCs.

### Inhibition of SGK3 attenuates liver CSCs expansion

**Fig. 3:**
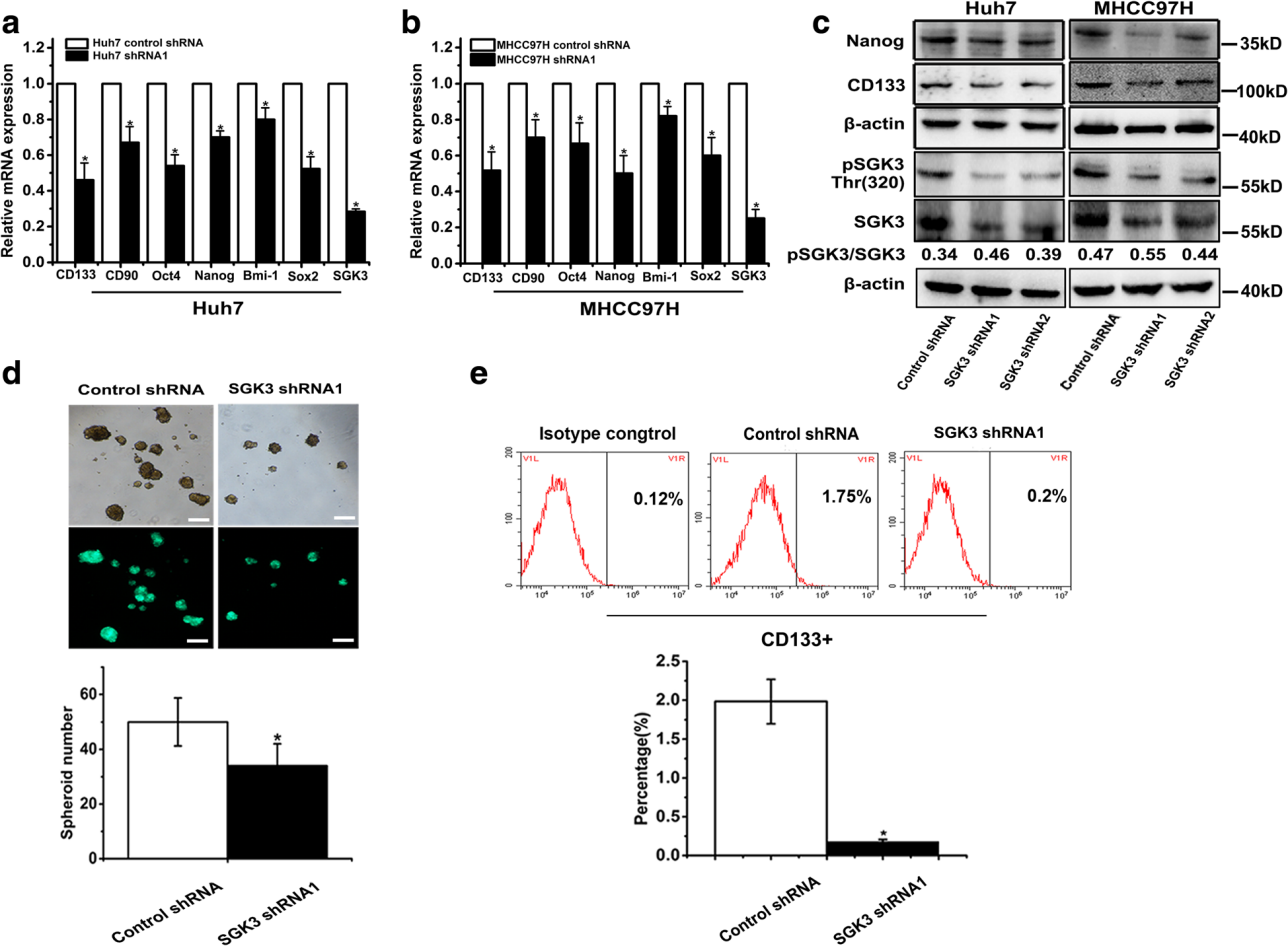
Inhibition of SGK3 attenuates liver CSC expansion. **a, b** Huh7-transfected SGK3 shRNA1 or MHCC97H-transfected SGK3 shRNA1 and their control cells were collected and subjected to real-time PCR. β-actin was used as a loading control. **c** Expression of Nanog, CD133, and SGK3 was detected by western blot in Huh7 shRNA1 or MHCC97H shRNA1 and their control cells. β-actin was used as a loading control. **d** Spheroid formation assay of SGK3 knockdown MHCC97H cells (MHCC97H-SGK3-shRNA1) or control cells (MHCC97H-SGK3-control) (up). Scale bars, 200 μm. The statistical results of the tumour spheroids (> 50 μm) were calculated based on 3 independent experiments (down). **e** The proportion of CD133+ cells in SGK3 knockdown MHCC97H cells or control cells was evaluated by flow cytometric assay. All experiments were performed in triplicate, and the results are shown as mean ± standard deviation. *P < 0.05

To further confirm the involvement of SGK3 in liver CSC expansion, 2 lentivirus vectors were designed to express shRNA for SGK3 knockdown (SGK3 shRNA1 and SGK3 shRNA2). After SGK3 inhibition, stemness genes were significantly suppressed in HCC cell lines (Fig. 3a and b). The protein levels of CD133 and Nanog were decreased in HCC cells after knockdown of SGK3, determined by western blot (Fig. 3c). In addition, SGK3 inhibition resulted in smaller spheroids, and they were significantly decreased in numbers (Fig. 3d). To confirm the result, flow cytometric analysis revealed a diminished proportion of liver CSCs in SGK3 shRNA1 stably transfected HCC cells (Fig. 3e).

### Prolonged treatment of HCC cells with PI3K inhibitors leads to activation of SGK3 and expansion of liver CSCs

**Fig. 4:**
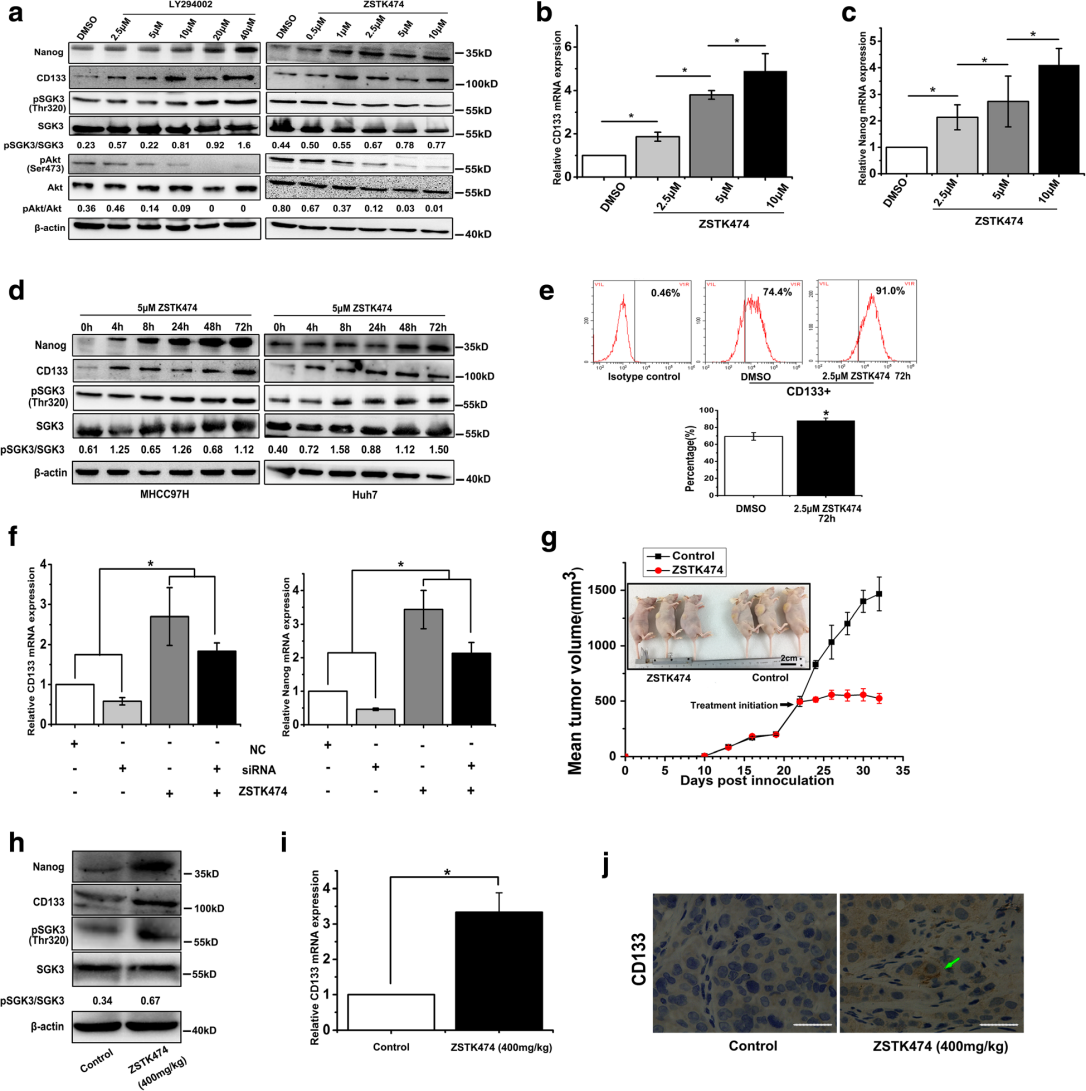
Prolonged treatment of HCC cells with PI3K inhibitors leads to activation of SGK3 and expansion of liver CSCs. **a** MHCC97H cells were treated with either LY294002 (concentration of 2.5, 5,10, 20, 40 μM) or ZSTK474 (concentration of 0.5, 1, 2.5, 5, 10 μM) for 24 h. Dimethyl sulphoxide (DMSO) was used as the control. Cell lysates were subjected to western blot analysis with the indicated antibodies. **b** Expression of CD133 was detected by RT-PCR in MHCC97H cells treated with ZSTK474 for 24 h. **c** Expression of Nanog was detected by RT-PCR in MHCC97H cells treated with ZSTK474 for 24 h. **d** Huh7 and MHCC97H cells were treated with 5 μM of ZSTK474 for 0 to 72 h. Cell lysates were subjected to western blot analysis with the indicated antibodies. **e** The proportion of CD133+ cells in Huh7 cells was evaluated by flow cytometric assay. **f** The suppression of SGK3 by siRNA in MHCC-97H cells treated with 5 μM of ZSTK474 resulted in a rescue of CD133 and Nanog overexpression, detected by RT-PCR. **g** The effect of the Class I PI3K inhibitor ZSTK474 on the growth of MHCC97H cells. **h** The protein levels of Nanog, CD133, pSGK3 and SGK3 in MHCC97H tumour xenografts in control and ZSTK474-treated mice. **i** The mRNA levels of CD133 in MHCC97H tumour xenografts in control and ZSTK474-treated mice. **j** Immunohistochemical staining of CD133 in the xenografted tumour xenografts treated with ZSTK474 or control. Scale bars, 20 μm. *P < 0.05

Because SGK3 can be activated by PI3K independent of AKT, we tested whether the phosphorylation of SGK3 can be blocked by PI3K inhibitors. We treated Huh7 cells with PI3K inhibitors (LY294002 and ZSTK474) for 24 h with increasing doses. Interestingly, the results showed that both inhibitors induced a dose-dependent increase in SGK3 phosphorylation, while the phosphorylation of Akt was inhibited with increasing concentration (Fig. 4a). In addition, after PI3K inhibitor treatment, we observed a dose-dependent increase in the expression of CSC-relative genes CD133 and Nanog (Fig. 4a, b and c). A recent study reported that prolonged treatment of breast cancer cells with class I PI3K or Akt inhibitors leads to increased expression and activation of SGK3 [20]. To investigate whether prolonged treatment of liver cancer cells with PI3K inhibitors could promote expression and activation of SGK3, we treated cells with ZSTK474 in a time gradient (0 h, 4 h, 8 h, 24 h, 48 h, and 72 h). As expected, prolonged ZSTK474 treatment over 24 to 72 h led to a time-dependent increase of SGK3 phosphorylation under conditions in which the CSC-relative genes CD133 and Nanog were induced (Fig. 4d).

Flow cytometric analysis revealed an enlarged proportion of CD133+ cells after Huh7 cells were treated with ZSTK474 for 72 h (Fig. 4e). Interestingly, knockdown of SGK3 expression employing siRNA partially blocked prolonged PI3K inhibitor treatment from enhancing CD133 expression in both Huh7 and MHCC97H cells (Fig. 4f). These results indicate that prolonged treatment with PI3K inhibitors induced expansion of CD133+ cells via enhancing SGK3 phosphorylation.

We next assessed the effect of ZSTK474 on tumour growth in an in vivo experiment. After the tumours formed (300–500 mm^3^), the mice were orally administered with 400 mg/kg of ZSTK474 for 10 days. The results indicate that ZSTK474 showed significant anti-tumour activity in the treatment period as compared to control mice (Fig. 4g). Western blot analysis of ZSTK474-treated tumours exhibited increased expression of Nanog and CD133, and the increased phosphorylation of SGK3 was validated (Fig. 4h). The increased mRNA level of CD133 was also confirmed in vivo (Fig. 4i). Immunohistochemical assay showed that tumours treated with ZSTK474 have higher levels of CD133 than control tumours (Fig. 4j).

### The inhibitor of hVps34 can block SGK3 activity and suppress liver CSC expansion induced by PI3K inhibitors

**Fig. 5:**
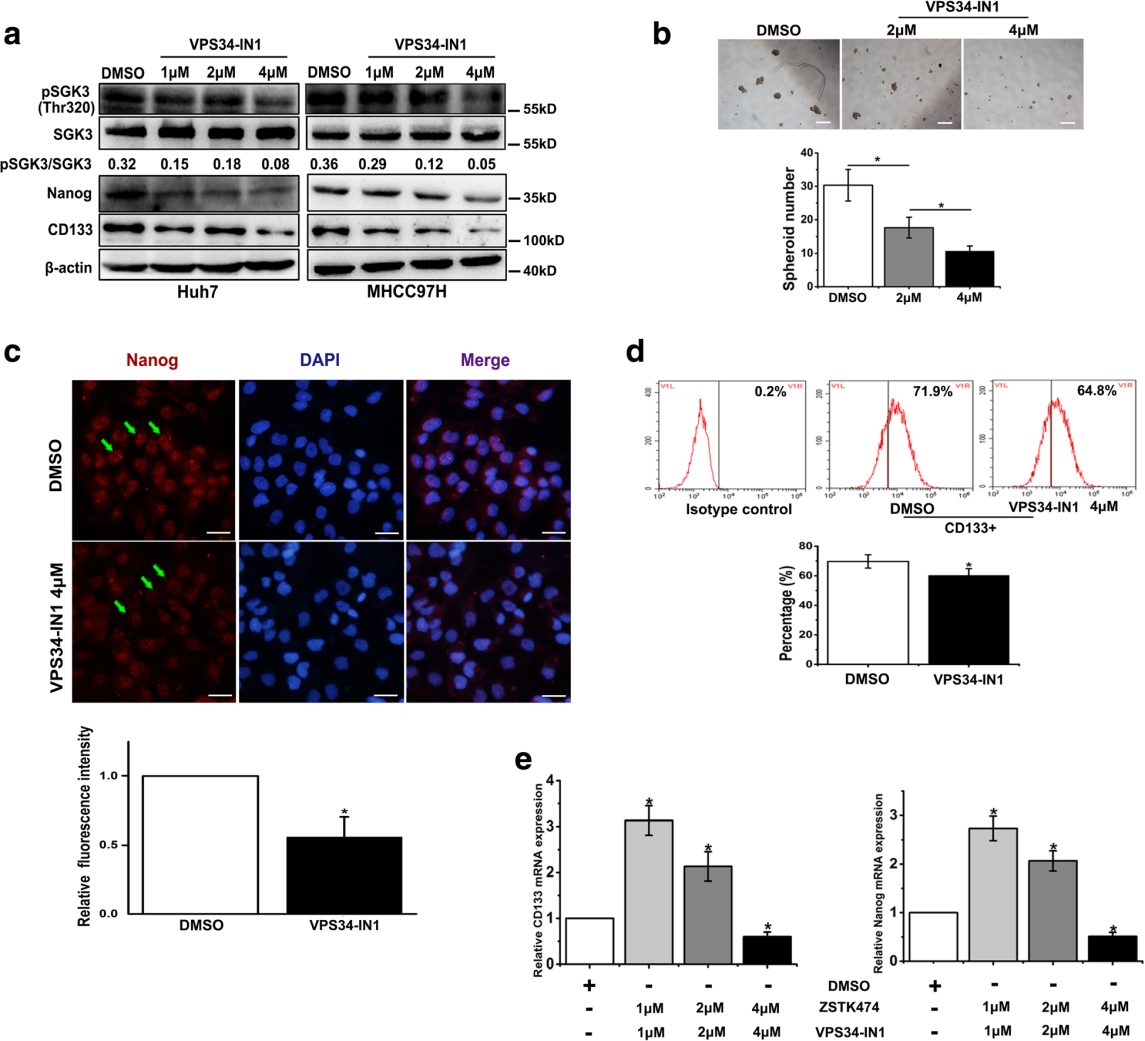
The inhibitor of hVps34 can block SGK3 activity and suppress liver CSC expansion after prolonged treatment of HCC cells with PI3K inhibitors. **a** Huh7 and MHCC97H cells were treated with VPS34-IN1 for 24 h, and cell lysates were subjected to western blot analysis with the indicated antibodies. **b** Spheroid formation assay of MHCC97H cells treated with VPS34-IN1 (top), the statistical results of the tumour spheroids (> 50 μm) were calculated based on 3 independent experiments (bottom). Scale bars, 200 μm. **c** Immunofluorescence images of Nanog (red) in MHCC7H cells after treatment with VPS34-IN1 or dimethyl sulphoxide DMSO for 24 h. Nuclei were stained with DAPI (blue). Scale bars, 10 μm. **d** The proportion of CD133+ cells in Huh7 cells was evaluated by flow cytometric assay. **e** Treatment of MHCC97H cells with VPS34-IN1 for 24 h inhibited the overexpression of CD133 and Nanog induced by treatment with ZSTK474, detected by RT-PCR. *P < 0.05

It has been reported that SGK3 can be activated by PtdIns(3)P produced by hVps34, and hVps34 inhibitor VPS34-IN1 can inhibit SGK3 activation [20, 21]. Our results confirmed that VPS34-IN1 induced a dose-dependent inhibition of SGK3 phosphorylation in MHH97H and Huh7 cells (Fig. 5a). In addition, treatment of Huh7 and MHCC97H cells with VPS34-IN1 also reduced the expression of CSC-related markers CD133 and Nanog (Fig. 5a and c). Consistently, hVps34 inhibitor VPS34-IN1 suppressed the spheroid formation ability tested by spheroid formation assay (Fig. 5b). Flow cytometric analysis revealed a diminished proportion of CD133+ cells after Huh7 cells were treated with VPS34-IN1 for 24 h (Fig. 5d). Furthermore, the increased expression of CD133 and Nanog induced by prolonged PI3K inhibitor treatment can be depleted by VPS34-IN1 (Fig. 5e). Collectively, these results indicated that the inhibitor of hVps34 can block liver CSC expansion after prolonged treatment of HCC cells with PI3K inhibitors via the inhibition of SGK3.

### SGK3 promotes liver CSCs through β-catenin accumulation by GSK3β

**Fig. 6:**
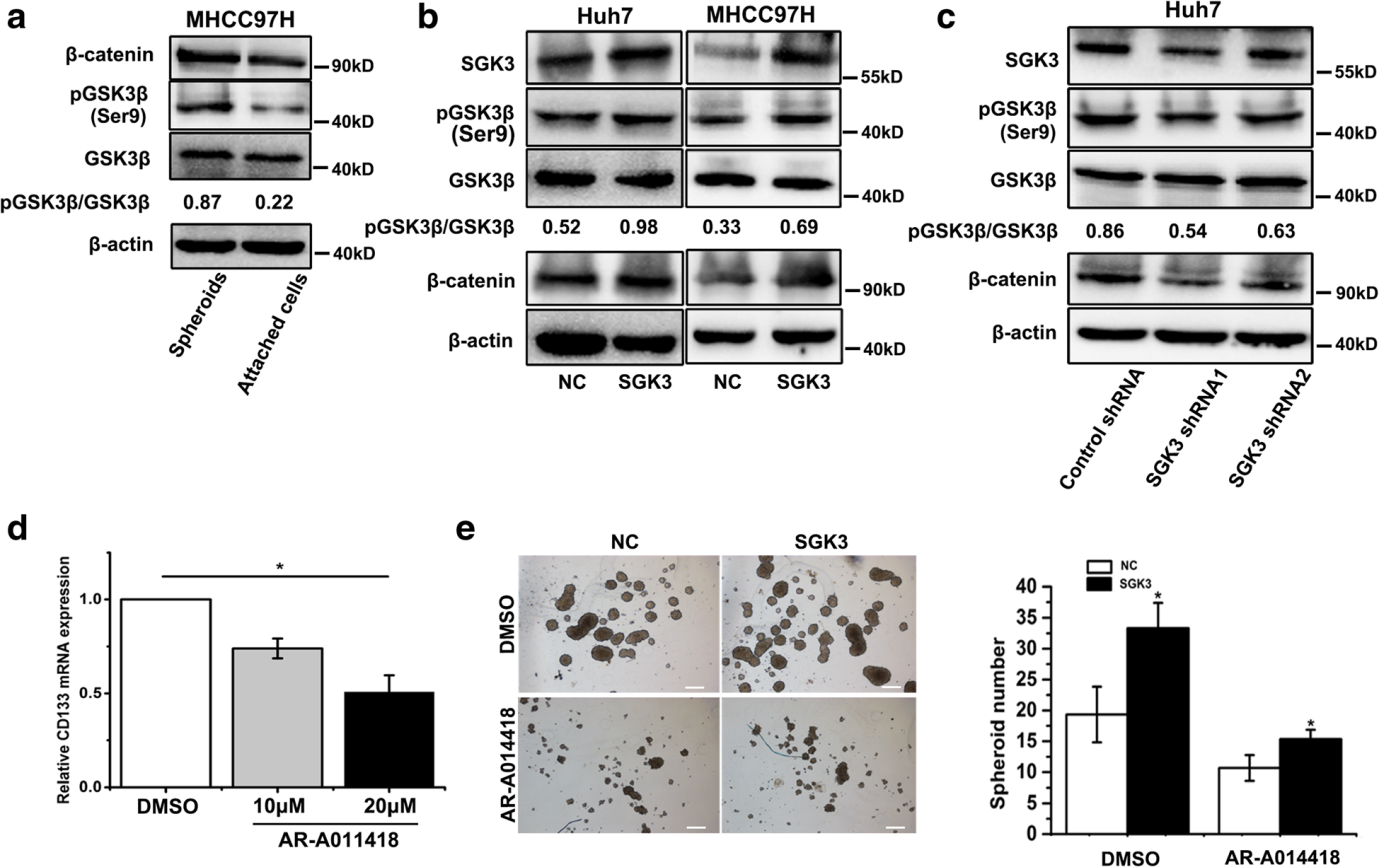
SGK3 promotes liver CSCs through β-catenin accumulation by GSK3β inactivation. **a** Levels of β-catenin and active GSK3β (phosphorylated at Ser9)/total GSK3β compared between MHCC97H spheroids and attached cells detected by western blot. β-actin was used as a loading control. **b** Expression of β-catenin, active GSK3β (phosphorylated at Ser9)/ total GSK3β and SGK3 in Huh7 and MHCC-97H cells stably overexpressing SGK3 or NC detected by western blot. β-actin was used as a loading control. **c** Expression of β-catenin, active GSK3β (phosphorylated at Ser9)/total GSK3β and SGK3 in Huh7 SGK3 shRNA cells or control cells was detected by western blot. **d** Treatment of MHCC97H cells with AR-A014418 for 24 h inhibited the expression of CD133, detected by RT-PCR. **e** MHCC97H-SGK3 or MHCC-97H-NC were treated with 20 μM of GSK3β inhibitor AR-A014418 and subjected to spheroid formation assay (left). The statistical results of the tumour spheroids (> 50 μm) were calculated based on 3 independent experiments (right). Scale bars, 200 μm. All experiments were performed in triplicate, and the results are shown as the mean ± standard deviation. *P < 0.05

Our previous study confirmed that SGK3 stimulates β-catenin signalling in HCC cells [23]. It is well established that SGK3 promotes the inactivation of GSK-3β by phosphorylation of GSK-3β on Ser9 [22]. To investigate whether SGK3 can promote liver CSC expansion via GSK-3β/β-catenin signalling, we first detected the expression of GSK-3β and β-catenin in spheroids and attached cells. The western blot analysis revealed that the phosphorylation of GSK-3β on Ser9 and expression of β-catenin was significantly increased in spheroid cells (Fig. 6a). We next analysed the phosphorylation of GSK-3β on Ser9 after SGK3 overexpression or knockdown. Our results indicated that overexpression of SGK3 increased the phosphorylation level of GSK-3β on Ser9 (Fig. 6b). Conversely, knockdown of SGK3 reduced the phosphorylation level of GSK-3β on Ser9 (Fig. 6c). Furthermore, overexpression of SGK3 increased β-catenin levels, whereas knockdown of SGK3 reduced β-catenin expression (Fig. 6b and c), indicating a canonical regulation of β-catenin stability by SGK3. Treatment of MHCC97H cells with AR-A014418, a selective GSK-3β inhibitor reduced the expression of CD133 (Fig. 6d). To further determine the role of SGK3 in liver CSC self-renewal via the GSK-3β/β-catenin signalling pathway, SGK3 overexpression and control cells were treated with AR-A014418. Spheroid formation analysis showed that AR-A014418 weakened the SGK3-enhanced self-renewal of liver CSCs (Fig. 6e).

### Prolonged treatment of HCC cells with PI3K inhibitors stimulates the β-catenin signalling pathway via activation of SGK3

**Fig. 7:**
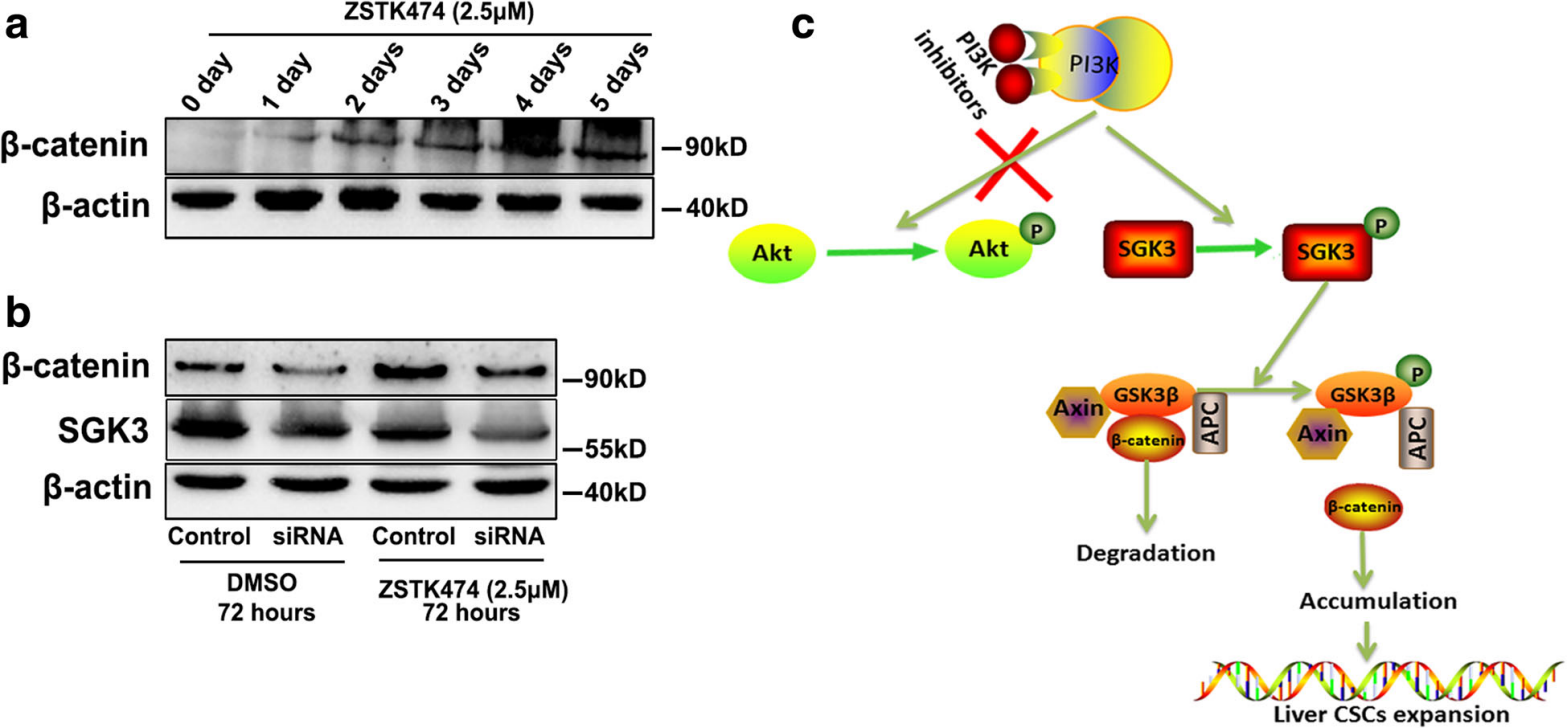
Prolonged treatment of HCC cells with PI3K inhibitors stimulates the β-catenin signalling pathway via activation of SGK3. **a** The expression of β-catenin in MHCC97H cells treated with 2.5 μM of ZSTK474 for 0 to 5 days was detected by western blot. **b** Western blot analysis of the effect of SGK3 knockdown by siRNA on the induced overexpression of β-catenin by treatment with 2.5 μM ZSTK474. **c** Schematic representation of prolonged treatment of HCC cells with class I PI3K inhibitors promotes the expansion of liver CSCs, partially by activating the SGK3/GSK3β/β-catenin signalling pathway. All experiments were performed in triplicate, and the results are shown as mean ± standard deviation. *P < 0.05

Nuclear accumulation of β-catenin has been reported to confer resistance to PI3K inhibitors in colon cancer [27]. To explore the β-catenin levels changed under prolonged treatment of HCC cells with PI3K inhibitors, we treated MHCC97H cells with PI3K inhibitors for 0 to 5 days and detected β-catenin expression. The results showed that prolonged PI3K inhibitor ZSTK474 treatment over 1 to 5 days led to a time-dependent accumulation of β-catenin (Fig. 7a). To study the effect that PI3K inhibitors had on β-catenin accumulation via SGK3, SGK3 was knocked down in MHCC97H cells by siRNAs and subjected to treatment with ZSTK474. As expected, inhibition of SGK3 decreased the accumulation of β-catenin after treatment with ZSTK474 for 72 h (Fig. 7b). Taken together, these data suggest that prolonged treatment of HCC cells with inhibitors of PI3K leads to marked accumulation of β-catenin via the activation of SGK3 (Fig. 7c).

## Discussion

CSCs have stem characteristics such as self-renewal, differentiation and tumourigenesis. CSCs have been recognised to contribute to cancer relapse and metastasis due to their invasive and drug-resistant capacities. It is therefore important to explore the molecular mechanism underlying liver CSC regulation so as to develop novel therapeutic strategies eliminating CSCs. In this study, we report that SGK3 plays a pivotal role in liver CSC expansion via the GSK3β/β-catenin signalling pathway, and the activation of SGK3 may contribute to the liver CSCs tolerance of PI3K inhibitor treatment.

SGK3 is emerging as a tumour oncogene in several cancers [28, 29]. Amplification and overexpression of SGK3 was frequently detected in HCC specimens, and SGK3 can promote HCC cell survival, proliferation and tumour formation in nude mice [22]. Our previous study found that SGK3 promotes HCC cell migration and invasive potential [23], which confirmed its vital function in promoting HCC progression. However, whether SGK3 also plays an important role in CSCs has not yet been reported. Our data showed a high phosphorylation level of SGK3 in liver CSC-enriched spheroids and CD133+ cells. More intriguingly, we demonstrated that overexpression of SGK3 enhances the expansion of liver CSCs, while inhibition of SGK3 by shRNA had an opposite effect. These findings indicate that SGK3 is critical for liver CSC expansion.

PI3K activity is stimulated by diverse oncogenes and growth factor receptors, and elevated PI3K signalling is considered a hallmark of cancer. Many PI3K pathway-targeted therapies have been tested in oncology trials [30]. Class I PI3K activates downstream effectors by generating phosphoinositides PtdIns-3, 4-P2 and PtdIns-3, 4, 5-P3. The shared property of these PI3K effectors is a pleckstrin homology (PH) domain selective for PtdIns-3, 4, 5-P3 or PtdIns-3, 4-P2 [30, 31]. Arguably, the vast majority of studies have focused on the protein kinase AKT as the dominant effector of PI3K signalling associated with malignancy. SGK3 has been reported to be a critical effector of oncogenic PIK3CA mutant breast cancer cells in which Akt is dispensable [17]. Although SGK3 lacks a PH domain, it may still be activated by Class I PI3K through PDK1 [29]. In contrast to Akt, SGK3 possesses an N-terminal PtdIns(3)P-binding PX domain [20, 32], which is predominantly produced at the endosome by the Class III PI3K hVps34. In the present study, we demonstrated that prolonged treatment with PI3K inhibitors triggers SGK3 activation in HCC cells, while the phosphorylation of Akt is inhibited, which is consistent with findings reported by Bago et al. [20]. We reasoned that the activation of SGK3 may be a compensatory mechanism to substitute the blockade of the Akt signalling pathway, which causes HCC cells to tolerate the treatment of PI3K inhibitors.

In the present study, we also found that prolonged treatment with PI3K inhibitors induced expansion of liver CSCs in HCC cells. We speculated that the expansion of liver CSCs induced by prolonged treatment with PI3K inhibitors occurred via the activation of SGK3. To further confirm this hypothesis, a rescue experiment was performed using SGK3 siRNA. The results indicated that knockdown of SGK3 expression employing siRNA partially blocked prolonged PI3K inhibitor treatment from enhancing CD133 expression in both Huh7 and MHCC97H cells. Meanwhile, our in vivo models confirmed that decreased tumour growth would be achieved by ZSTK474 treatment. Consistent with the in vitro experiment, ZSTK474 treatment induced the expansion of liver CSCs and activation of SGK3 in vivo. Although ZSTK474 could inhibit the tumor growth via inhibit proliferation and promote apoptosis of non-stem cells, a small proportion of cells could survive via their owned or acquired stemness property. Inhibition of mTOR signalling has been reported to upregulate CD133 expression in gastrointestinal cancer cells [33]. Because the PI3K signalling pathway is a complex regulatory network, other downstream signal molecules may play a role in the expansion of liver CSCs induced by prolonged treatment with PI3K inhibitors. Class I PI3K is known to negatively regulate autophagy via the AKT-MTORC1-ULK1 complex [34]; thus, autophagy other than SGK3 may also be involved in regulating the expansion of liver CSCs after prolonged treatment with PI3K inhibitors in HCC cells.

By virtue of its PX domain, SGK3 can bind to PtdIns(3)P produced by hVps34 on the endosome; thus, hVps34 inhibitor can inhibit SGK3 activation. Our results confirmed that VPS34-IN1, an hVps34 inhibitor, induced a dose-dependent inhibition of SGK3 phosphorylation. Furthermore, the inhibition of hVps34 also causes the inhibition of liver CSC self-renewal. These results further confirm that SGK3 is critical for liver CSC expansion and that targeting SGK3 could be a promising strategy for HCC therapy.

The augmentation of Wnt/β-catenin signalling through Ser9 phosphorylation-inactivation of GSK3β is a well-recognised regulatory pathway for CSC self-renewal and cancer development [35, 36]. Our previous study confirmed that SGK3 stimulates β-catenin signalling in HCC cells [23]. Liu et al. reported that overexpression of SGK3 increased the phosphorylation level of GSK3-β on Ser9 and inactivates GSK3-β [22]. In our present study, we further confirmed that SGK3 promotes β-catenin accumulation by increasing the phosphorylation level of GSK3-β on Ser9, inactivating GSK3-β and inhibiting the degradation of β-catenin. Importantly, our results demonstrated that SGK3 promotes liver CSC expansion through the GSK3β/β-catenin signalling pathway. It has been previously reported that nuclear accumulation of β-catenin confers resistance to PI3K inhibitors in colon cancer [27]. Indeed, our data suggested that prolonged inhibition of Class I PI3K leads to significant accumulation of β-catenin. We speculated that the accumulation of β-catenin was through the activation of SGK3 in HCC cells subjected to prolonged inhibition of Class I PI3K, which led to the enhanced self-renewal of liver CSCs.

## Conclusions

In summary, our findings reveal that SGK3, a novel oncogene, plays a vital role in the expansion of liver CSCs through the GSK3β/β-catenin signalling pathway. Prolonged treatment of HCC cells with class I PI3K inhibitors promotes the expansion of liver CSCs, partially by activating the SGK3/ GSK3β/β-catenin signalling pathway (Fig. 7c). However, there is no specific inhibitor to SGK3. Perhaps, the development of this inhibitor will effectively eliminate the CSCs and improve the effect of PI3K inhibitors in the treatment of cancers.

## Additional file

Additional file 1: **Table S1.** Primer sequences for quantitative RT-PCR. (DOCX 24 kb)

## Abbreviations

CSCs: Cancer stem cells
GSK3β: Glycogen synthase kinase 3β
HCC: Hepatocellular carcinoma
PI3K: Phosphoinositide 3-kinase
SGK3: Serum and glucocorticoid-regulated kinase 3
