# Supplementary material for: Prolonged inhibition of class I PI3K promotes liver cancer stem cell expansion by augmenting SGK3/GSK-3β/β-catenin signalling
Source: J Exp Clin Cancer Res. 2018 Jun 25;37:122. doi: 10.1186/s13046-018-0801-8 (PMC6020243; doi:10.1186/s13046-018-0801-8)
Supplement: Supplementary file 1 — Table S1. Primer sequences for quantitative RT-PCR. (DOCX 24 kb) [file 13046_2018_801_MOESM1_ESM.docx]

Additional file

Additional file 1**: Table S1** Primer sequences for quantitative RT-PCR

| Gene Forward (5’-3’) Reverse (5’-3’) |
| --- |
| SGK3 GCTGCCCAAGTGTAAGCATTC GTTTAGTCCTGCTCGTCTTTGTT  Nanog AATACCTCAGCCTCCAGCAGATG TGCGTCACACCATTGCTATTCTTC  Sox2 GCCGAGTGGAAACTTTTGTCG GGCAGCGTGTACTTATCCTTCT  Oct4 CTTGCTGCAGAAGTGGGTGGAGGAA CTGCAGTGTGGGTTTCGGGCA  CD90 GACCCGTGAGACAAAGAAGC GCCCTCACACTTGACCAGTT  CD133 TGGATGCAGACCTTGACAACGT ATACCTGCTACGACAGTCGTGGT  Bmi-1 TGGAGAAGGAATGGTCCACTTC GTGAGGAAACTGTGGATGAGGA  β-actin CCTGGCACCCAGCACAAT GGGCCGGACTCGTCATAC |
